# Psychosocial Intervention Is Associated with Altered Emotion Processing: An Event-Related Potential Study in At-Risk Adolescents

**DOI:** 10.1371/journal.pone.0147357

**Published:** 2016-01-25

**Authors:** Hannah L. Pincham, Donna Bryce, Danae Kokorikou, Peter Fonagy, R. M. Pasco Fearon

**Affiliations:** 1 Developmental Neuroscience Unit, The Anna Freud Centre, 12 Maresfield Gardens, London, United Kingdom, NW3 5SU; 2 Research Department of Clinical, Educational and Health Psychology, University College London, 1–19 Torrington Place, London, United Kingdom, WC1E 7HB; 3 Department of Psychology, University of Tübingen, Schleichstrasse 4, 72076, Tubingen, Germany; Chiba University Center for Forensic Mental Health, JAPAN

## Abstract

Emotion processing is vital for healthy adolescent development, and impaired emotional responses are associated with a number of psychiatric disorders. However, it is unclear whether observed differences between psychiatric populations and healthy controls reflect modifiable variations in functioning (and thus could be sensitive to changes resulting from intervention) or stable, non-modifiable, individual differences. The current study therefore investigated whether the Late Positive Potential (LPP; a neural index of emotion processing) can be used as a marker of therapeutic change following psycho-social intervention. At-risk male adolescents who had received less than four months intervention (minimal-intervention, N = 32) or more than nine months intervention (extended-intervention, N = 32) passively viewed emotional images whilst neural activity was recorded using electroencephalography. Significant differences in emotion processing, indicated by the LPP, were found between the two groups: the LPP did not differ according to valence in the minimal-intervention group, whereas the extended-intervention participants showed emotion processing in line with low risk populations (enhanced LPP for unpleasant images versus other images). Further, an inverse relationship between emotional reactivity (measured via the LPP) and antisocial behaviour was observed in minimal-intervention participants only. The data therefore provide preliminary cross-sectional evidence that abnormal neural responses to emotional information may be normalised following psychosocial intervention. Importantly, this study uniquely suggests that, in future randomised control trials, the LPP may be a useful biomarker to measure development and therapeutic change.

## Introduction

A crucial aspect of adolescent development concerns the successful maturation of emotional responding. Given that self-report measures of emotion perception are often unreliable, physiological techniques are the method of choice for investigating normal and abnormal emotion processing [1, 2]. Difficulties with the processing of emotions are key features of a wide variety of psychological and behavioural disorders in children and adults. For example, emotional reactivity is altered (as measured using fMRI, startle responses and EEG, see [3–5]) in depression. Similarly, reduced emotionality appears to be a critical feature of antisocial problems and conduct disorder [6, 7]. The Late Positive Potential (LPP) is frequently employed in developmental research environments as a robust index of emotion processing in young people. The LPP is an electrophysiological measure that is larger (more positive) following the viewing of affective images compared with neutral images. This component appears to be sensitive to emotional and behavioural problems in young people. For example, we recently demonstrated that emotion processing (measured via the LPP) was reduced in teenage males involved in criminal activities versus their non-offending peers [1]. The LPP may therefore be a good biomarker of disturbed emotional functioning and of emotional wellbeing. This could be particularly useful in children and adolescents, who may find it difficult to verbalise emotional content. However, whether the LPP could be employed to assess *changes* in emotion processing associated with psychological treatment or other therapeutic interventions remains an important, but open, question. In the current study, we aimed to provide initial evidence regarding the sensitivity of the LPP as a marker of therapeutic change by examining whether emotion processing differed between two groups of young people involved in a community-based psychosocial program. Specifically, we measured emotion processing in at-risk adolescents who were relatively new to the psychosocial program, versus those who had been involved in the program for approximately one year. Although this study design cannot assess intra-individual changes in emotion processing, the groups were well-matched on a range of factors. Thus, demonstrating reliable LPP differences would provide support for the potential value of the LPP as a biomarker of therapeutic effectiveness in young people that could be tested in future randomised control trial (RCT) studies. More generally, the identification of reliable biomarkers of psychopathology and of treatment-change is critical for advancing translational science, particularly within developmental contexts.

It is important to note that previous investigations of emotion processing in adolescents (including our own) have typically contrasted psychiatric populations with healthy control participants. Consequently, it is unclear in these studies whether the LPP is tracking current functioning (and hence may be sensitive to change in functioning), or whether it reflects static individual differences or stable risk factors that differ between psychiatric groups and healthy controls (and hence may not change as a function of treatment). In a novel contribution to the field, the current study addresses this issue by asking whether emotion processing differs as a result of involvement in a psychosocial intervention program. Although the ideal research design for testing such effects is a longitudinal RCT, such methods are highly costly. As a first step in establishing the potential value of measuring electrophysiological indices of treatment effects, we examine whether differences in emotion processing exist between the two groups of adolescents involved in a psychosocial intervention who differ in the amount of treatment they have received–the first group was new to the programme and had received minimal therapeutic intervention, and the second was a group of adolescents who had been assigned a therapeutic keyworker and had received at least 9 months of intervention. Reliable differences between these groups would provide important ‘proof of principle’ evidence that the LPP may be sensitive to therapeutic change.

The community intervention programme that forms the basis of this study involved a group of young people experiencing multiple social adversities. In common with many other developmental studies of community interventions, these ‘at-risk’ youth do not represent a distinct psychiatric population. However, these young people typically live in high stress environments characterised by poverty, community violence and trauma [8], and present with a range of emotional and behavioural difficulties, particularly conduct problems and antisocial behaviour. Emotional impairments in such young people are perhaps not surprising, given that poverty and chronic adversity are empirically associated with disruptive behaviour and poor psychological well-being [9, 10]. Therefore, emotional processing is a logical target system for measuring biological processes associated with psychosocial intervention for at-risk youth. Consistent with that line of reasoning, physiological research indicates that externalizing behavioural problems are specifically linked to problems with emotion processing, and particularly with reduced emotional reactivity. Children with a diagnosis of conduct disorder, for example, demonstrate reduced autonomic activity to aversive stimuli [2, 11–13], as do adolescents later convicted of crime [14]. Neuroimaging data also support the notion that emotional hypo-reactivity is associated with externalizing behaviour. Neural hypo-activation has been reported in brain regions typically implicated in emotion processing, including the amygdala, the anterior cingulate cortex and the anterior insula during affective image processing, facial expression processing and emotional decision making [2, 7, 12]. Considering that psychosocial interventions, including the programme considered here, aim to improve cognitive, social and emotional functioning in young people, we hypothesised that emotion processing (indexed via the LPP) would differ between the two groups in our study. In particular, if, as expected, the programme leads to improvements in emotional processing, we would expect adolescents who had received extensive psychosocial support to have more normative LPP responses to emotion-eliciting stimuli than those who had yet to receive extensive support.

In this study we examine two neural indices of emotion processing: the early posterior negativity (EPN) and the late positive potential (LPP). The EPN is one of the earliest components responsive to emotional material (beginning approximately 150msec after stimulus onset) and is larger (more negative) for arousing, compared with neutral, stimuli [15–17]. The latency and bilateral temporo-occipital topography of the EPN suggest that it is generated in the visual cortex, and is an index of early visual perception and attention [18]. In contrast, the LPP onsets around 400msec and is reliably larger (more positive) for arousing stimuli than for neutral stimuli [19–21]. The LPP has been argued to reflect a higher-order obligatory motivational system [22], rather than early perception or the evaluative categorisation of stimuli [20, 23, 24]. This explanation is consistent with the LPP’s proposed neural generators, which comprise emotional and motivational brain circuits including the amygdala, insula and cingulate cortex [25]. Consistent with this interpretation of the EPN and LPP, we recently found evidence indicating that only the later measure of emotion processing was impaired in juvenile offenders [1]. In this study, although the EPN of all participants was responsive to the emotional content of the images, the LPP was modulated by emotion for control participants but not juvenile offenders. Specifically, offenders demonstrated neural hypo-reactivity to unpleasant images, indicating that late emotion processing was suppressed in those participants. We thus advanced the hypothesis that those involved in the intervention programme for longer will show larger LPPs than those new to the programme. The EPN, by contrast, is not expected to show differences, thereby providing evidence that any LPP differences are not a result of differences in lower-level attention.

Two groups of participants were drawn from a psychosocial program provided by a charity based in London, UK (Kids Company). The intervention is based on principles derived from attachment theory, and involves an at-risk young person being assigned to an adult key-worker who provides practical help and emotional support [26, 27]. The intervention consists of support in five areas: educational (for example, providing support for the client to enrol in school or college, and helping them to remain in school and avoid school exclusion), emotional (for example, providing a safe and confidential environment for the client to express their concerns, or overseeing the provision of formal therapeutic services), housing (for example, assisting the young person to find safe and stable living arrangements), legal (for example, assisting the young person with immigration concerns, benefit claims or in navigating the criminal justice system) and physical health (for example, visits to the gym, or providing sexual health information). In common with other community-based interventions [28–30], this program was designed for children experiencing multiple chronic adversities (such as poverty), rather than children with a single, specific psychiatric or psychological disorder. The broad package of emotional and social support embedded in the youth-keyworker relationship aims to improve emotional and social well-being, tackle practical problems with housing and schooling, enhance attachment, promote empathy, and assist the young person to better deal with stress.

## Materials & Methods

### Participants

Participants were 80 male adolescents (aged 13–18 years). All participants were initially assessed by charity staff as being ‘at-risk’. To qualify as at-risk, the following criteria must have been met: the individual displayed emotional turmoil, serious risk-taking behaviours, homelessness, criminal behaviour, signs of neglect and/or abuse [31]. Of these participants, 9 were excluded due to poor EEG data quality. Given that ERP amplitudes are strongly correlated with age, we carefully matched participant ages across the minimal-intervention and extended-intervention groups, excluding 15 participants that could not be age-matched across the groups. This resulted in 32 participants per group.

All participants in the minimal-intervention group had received less than 4 months intervention (mean = 1.79, SD = 1.55, range = 0–3.87), and all participants in the extended-intervention group had received more than 9 months intervention (mean = 16.57, SD = 8.07, range = 9.4–36.13). For the extended-intervention group, all participants had engaged in the intervention program on a regular basis. Degree of engagement in the program was rated by participants’ key-workers.

Socio-economic status was determined using a freely available tool from the Office for National Statistics (UK). This tool provides estimates for the percentage of households living in poverty, where poverty is defined as living below 60% median income. The estimates are derived using survey, census and administrative data in order to produce accurate data for small geographic area statistics. As shown in Table 1, the minimal-intervention and extended-intervention groups did not differ in age or socio-economic status.

**Table 1:** Demographic and behavioural characteristics (mean and standard deviations) for the minimal-intervention and extended-intervention groups (N = 32 per group).

		Minimal-Intervention	Extended-Intervention	p-value	Effect size
Age		15.28 (1.23)	15.39 (1.27)	0.729	.088
Households in Poverty (%)		32.53 (7.87)	33.22 (10.13)	0.762	.078
SRYB		1.84 (0.49)	1.82 (0.47)	0.913	.028
SDQ					
	*Pro-Social Behaviour*	7.38 (2.01)	7.78 (1.74)	0.391	.220
	*Hyperactivity*	4.88 (2.52)	4.97 (2.86)	0.890	.035
	*Emotional Symptoms*	2.78 (1.98)	2.44 (1.81)	0.472	.184
	*Conduct Problems*	3.63 (2.01)	2.63 (2.03)	0.052	.503
	*Peer Problems*	2.28 (1.61)	2.38 (1.74)	0.397	.057
KW Ratings					
	*Internalising behaviour*	2.14 (0.89)	2.44 (0.72)	0.175	.068
	*Externalising behaviour*	2.00 (0.93)	2.06 (0.91)	0.807	.382
	*Life Stressors*	1.90 (0.94)	2.28 (0.92)	0.156	.403

*Note*: The groups did not differ on any measure except for reduced conduct problems in the minimal-intervention group. Effect sizes using Cohen’s d for independent samples t-tests are shown.

The study was approved by the Research Ethics Committee at University College London (ID: 3064/001). Written, informed consent was provided by all participants, and was also obtained from parents or legal guardians for participants under 16 years of age, but not for participants aged over 16 years. The ethics committee specifically approved these consent procedures. Participants had normal or corrected-to-normal vision, and none had been diagnosed with a developmental disability. All participants were compensated with a £30 shopping voucher.

### Intervention

Psychosocial intervention was independently provided using the key-worker model detailed in the Introduction. Key-workers receive regular, on-going training in attachment theory, therapeutic engagement skills and other areas of clinical practice relevant for at-risk youth (for example, dealing with antisocial behaviour, responding to trauma, improving educational attainment).

Semi-structured interviews were conducted with each participant’s key-worker to characterise the participants’ initial emotional and behavioural difficulties. In this manner, we aimed to assess how well matched the two groups were prior to intervention. Key-workers were asked whether–prior to intervention–their client had been excluded from school due to poor conduct, had been involved in criminal activity or had experienced a trauma (for example, witnessed or been exposed to violence, physical, emotional or sexual abuse, maltreatment or neglect, bereavement of a close caregiver, raised in foster care). Key-workers were also asked to rate on a three-point scale, the degree to which–prior to intervention–their client exhibited externalising behaviours (for example, extreme aggression, conduct problems), internalising behaviours (for example, anxiety or depression), and whether they had major stressors impacting on their lives (for example, homelessness or extreme poverty). Ten participants in the minimal-intervention group had not yet been assigned to a key-worker. For these participants, assessment case file records were consulted in order to establish the presence or absence of the above intake characteristics. Missing scores were recorded if the case files were inconclusive. Key-workers of extended-intervention clients also described the nature of intervention received by their client and indicated which of the following types of intervention had been received: educational, emotional, housing, legal and/or physical health.

### Stimuli & Measures

#### Picture Viewing Task

The task consisted of three blocks of 69 images from the International Affective Picture System (IAPS), which equated to 207 stimuli in total [32]. In each block, 23 images were pleasant (for example, families, animals), 23 were unpleasant (for example, illness, aggression) and 23 were neutral (for example, household objects, transport). Pleasant images were the following IAPS images: 1441, 1920, 2080, 2154, 2303, 2306, 4007, 4250, 4622, 5210, 5700, 5760, 5780, 5825, 5833, 8080, 8190, 8300, 8370, 8380, 8420, 8490, 8510. Neutral images were the following IAPS images: 1560, 1670, 2032, 2038, 2122, 2271, 2309, 2411, 2487, 2575, 2749, 2880, 5740, 7021, 7036, 7092, 7461, 7476, 7496, 7632, 9210, 9331, 9411. Unpleasant images were the following IAPS images: 2095, 2205, 2345, 2375, 2800, 3180, 3301, 3350, 3500, 3530, 3550, 6313, 9000, 9001, 9050, 9183, 9325, 9421, 9520, 9560, 9901, 9910, 9925.

Only age-appropriate stimuli were used and images of erotica and mutilation did not form part of the stimulus set. Based on normative adult data, the pleasant images (mean valence = 7.56, SD = 0.48) were higher in valence than the neutral images (mean valence = 5.09, SD = 0.62; *p* < .001) and unpleasant images (mean valence = 2.47, SD = 0.38, *p* < .001), and the neutral images were higher in valence than the unpleasant images (*p* < .001). The pleasant (mean arousal = 5.34, SD = 1.23) and unpleasant images (mean arousal = 5.35, SD = 0.83) were rated as more arousing than the neutral images (mean arousal = 3.87, SD = 0.89; *p* < .001). Arousal did not differ between the pleasant and unpleasant images (*p* = .967). Although the normative values reflect adult responses, developmental research using the IAPS images has confirmed that children distinguish similarly between pleasant, unpleasant and neutral images [33]. Following completion of the three passive viewing blocks, participants rated the valence of each image using a 9-point scale. Participants were asked to rate each image for how much pleasure it gave them. To assist with this task, images of sad, neutral and happy faces were positioned above the beginning, middle and end points of the scale respectively.

Previous work has highlighted that the LPP can be driven by perceptual features of the stimuli, rather than valence or arousal per se [20, 34, 35]. We therefore matched images to ensure that any observed differences in the LPP were driven by stimulus meaning and not visual features of the task. The visual complexity of each image was categorised as either simple (figure-ground composition) or complex (scenes with no main focus). Forty-eight per cent of the unpleasant images, 52% of the neutral images, and 35% of the pleasant images were simple compositions. Furthermore, an attempt was made to match the groups of images for the number of images that featured people (78, 70, and 61%, respectively) and animals (10, 13, 10%, respectively).

Each image was presented once per block in a randomised order. Images were presented in colour on a Dell E2009W monitor and the task was programmed using E-Prime 2.0 software (Psychology Software Tools, Pittsburgh, PA). Images appeared on a black background, 10cm x 12cm in size, for 1000 msec with a variable inter-stimulus interval ranging between 2300–2700 msec. Each image was shown once per block (hence, three times over the entire experiment).

#### Strengths and Difficulties Questionnaire

Participants completed the Strengths and Difficulties Questionnaire [36]. This measure is suitable for use in community samples and provides sub-scale scores for pro-social behaviour, hyperactivity, emotional symptoms, conduct problems and peer problems.

#### Self-Report of Youth Behaviour

Antisocial behaviour was assessed using this validated questionnaire, which measures the prevalence and recent frequency of delinquent behaviours including vandalism, theft and fraud [37]. Three additional questions were added to ask about school exclusion, weapon use and police arrests. Based on the guidance from Jessor and Jessor [38], total scores were derived, which take into account whether each behaviour has ever been committed, as well as frequency of the behaviour in the last six months. Higher total scores are indicative of more frequent, recent involvement in criminal activities and/or delinquent behaviour.

### Experimental Procedure

The participants were administered the IAPS task and self-report questionnaires in one session in a therapy room within a youth centre complex. Using a portable EEG set-up, we were able to conduct these measurements in the community, rather than relying on university or hospital based equipment and testing facilities, which no doubt aided participation in this group of adolescents, who are notoriously difficult to engage in research. During the IAPS task, participants passively viewed images whilst EEG was recorded. Participants were asked to think about how the images made them feel. Blinking was encouraged during the inter-stimulus interval to avoid artifacts contaminating the data. Participants rated the images for pleasantness and completed the two self-report measures after the passive viewing task.

### EEG Acquisition and Pre-processing

EEG was recorded using the HGSN Electrical Geodesics system. The sampling rate was 250 Hz. An anti-aliasing low-pass filter of 70 Hz was applied during data acquisition. Offline, the data were band-pass filtered between 0.1 and 30 Hz and recomputed to an average reference. The continuous EEG was segmented into epochs between –200 and 2000 ms relative to the onset of each image. Spline interpolation was carried out on individual channels if required. For the minimal-intervention group, the mean percentage and range of interpolated channels was 2.90% (range: 0.78–7.81%). For the extended-intervention group, the mean percentage and range of interpolated channels was 2.83% (range: 0.78–6.25%). Independent component analysis was run using FASTER to remove stereotyped artifacts [39]. Epochs were excluded from analysis if they met either of the following artifact rejection criteria: voltage deviations exceeded 100μV relative to baseline or the peak to peak moving amplitude exceeded 100μV in a 200msec moving window.

### Data Analysis

The two groups were contrasted across each self-report measure using independent-sample t-tests. To confirm that the groups were initially matched for severity of need, key-worker ratings of internalising behaviours, externalising behaviours and life stressors were compared using independent samples t-tests. The proportion of participants who had experienced school exclusion, criminal engagement and trauma were compared across the groups using chi-square tests. Participants’ mean valence ratings for the images were compared using an image valence (pleasant vs. neutral vs. unpleasant) x group (pre- intervention vs. extended-intervention) ANOVA. For all behavioural and ERP analyses, post-hoc contrasts were corrected for multiple comparisons using the Sidak procedure, where required.

#### EPN

Using the same definition as Schupp, Junghöfer, et al. [24], the EPN was defined as the mean amplitude in the window 240-280msec after stimulus onset, in lateral temporo-occipital regions. These data were analysed using an ANOVA with hemisphere (left vs. right) and image valence (pleasant vs. neutral vs. unpleasant) as within-subjects factors and group (minimal-intervention vs. extended-intervention) as the between-subjects factor. The left lateral temporo-occipital region comprised electrodes 56, 63, 64 (P9), 65 (PO7), 66, 68, 69, 70 (O1), 73, 74. The right lateral occipito-temporal region comprised electrodes 82, 83 (O2), 84, 88, 89, 90 (PO8), 94, 95 (P10), 99, 107. All electrode numbers are taken from the Electrical Geodesics sensor net.

#### LPP

Following Hajcak and Dennis [40], mean LPP amplitudes were calculated in parietal-occipital scalp locations. Electrodes used were O1, O2, P3, P4, P7, P8, which correspond to electrodes 70, 83, 52, 92, 58, 96 in the Electrical Geodesics sensor net. Difference topographies confirmed that, in the current study, observed effects were strongest in this same parietal-occipital region. To examine neural processing across time, mean amplitudes were calculated at two consecutive time windows, as per Cuthbert, et al. [20]: 400-700msec and 700-1000msec. Mean amplitudes in each time window were analysed using a repeated-measures ANOVA, with hemisphere, image valence and group as factors.

Importantly, none of the ERP effects reported below could result from differences in the number of trials accepted across image valence categories or across groups. For the minimal-intervention group, the mean numbers of accepted epochs (out of a maximum of 69 per category) were 54.94 (unpleasant), 55.03 (neutral) and 52.78 (pleasant). For the extended-intervention group, the mean numbers of accepted epochs were 54.41 (unpleasant), 56.03 (neutral) and 55.66 (unpleasant). An image valence x group ANOVA confirmed that accepted trial counts were statistically equivalent across the valence categories and between the groups (largest *F* = 1.969).

## Results

### Sample Characteristics

The minimal-intervention and extended-intervention groups were matched for age and socio-economic status. According to key-worker ratings, the two groups were well matched prior to intervention. The groups appeared to have been similar based on key-worker retrospective ratings of internalising behaviours, externalising behaviours and severity of life stressors. Further, the groups were similar in terms of the percentage of participants who had been excluded from school (77.27% for minimal-intervention versus 68.75% for extended-intervention; *p* = .261), engaged in criminal activity (36.36% versus 46.88%; *p* = .443) or experienced trauma (80.95% versus 78.13%; *p* = .804).

Key-workers described the level of intervention received by extended-intervention participants in an interview: 78% had received educational support, 94% received emotional support, 56% received help with housing, 38% received legal support and 60% received physical health intervention. Current levels of emotional and behavioural difficulties (as indexed by participants’ self-report ratings) were similar across the groups. The exception to this was for the Conduct Problems sub-scale of the SDQ, where behavioural problems were reduced, as expected, in the extended-intervention group compared with the minimal-intervention group, although that difference fell just short of statistical significance (*t*(62) = 1.980, *p* = .052). Demographic and behavioural characteristics are presented in Table 1.

### Valence Ratings

As expected, ratings of pleasantness differed across the three picture categories (*F*(2,124) = 289.840, *p* < .001, *η*^*2*^ = .824). Post-hoc contrasts confirmed that pleasant images (mean = 6.78, SD = 0.90) were rated more highly than neutral images (mean = 4.90, SD = 0.52; *p* < .001) and unpleasant images (mean = 3.07, SD = 0.94; *p* < .001). Neutral images were rated more highly than unpleasant images (*p* < .001). There was no effect of group on valence ratings (all *F*s<1).

### ERP Results: EPN

**Fig 1:**
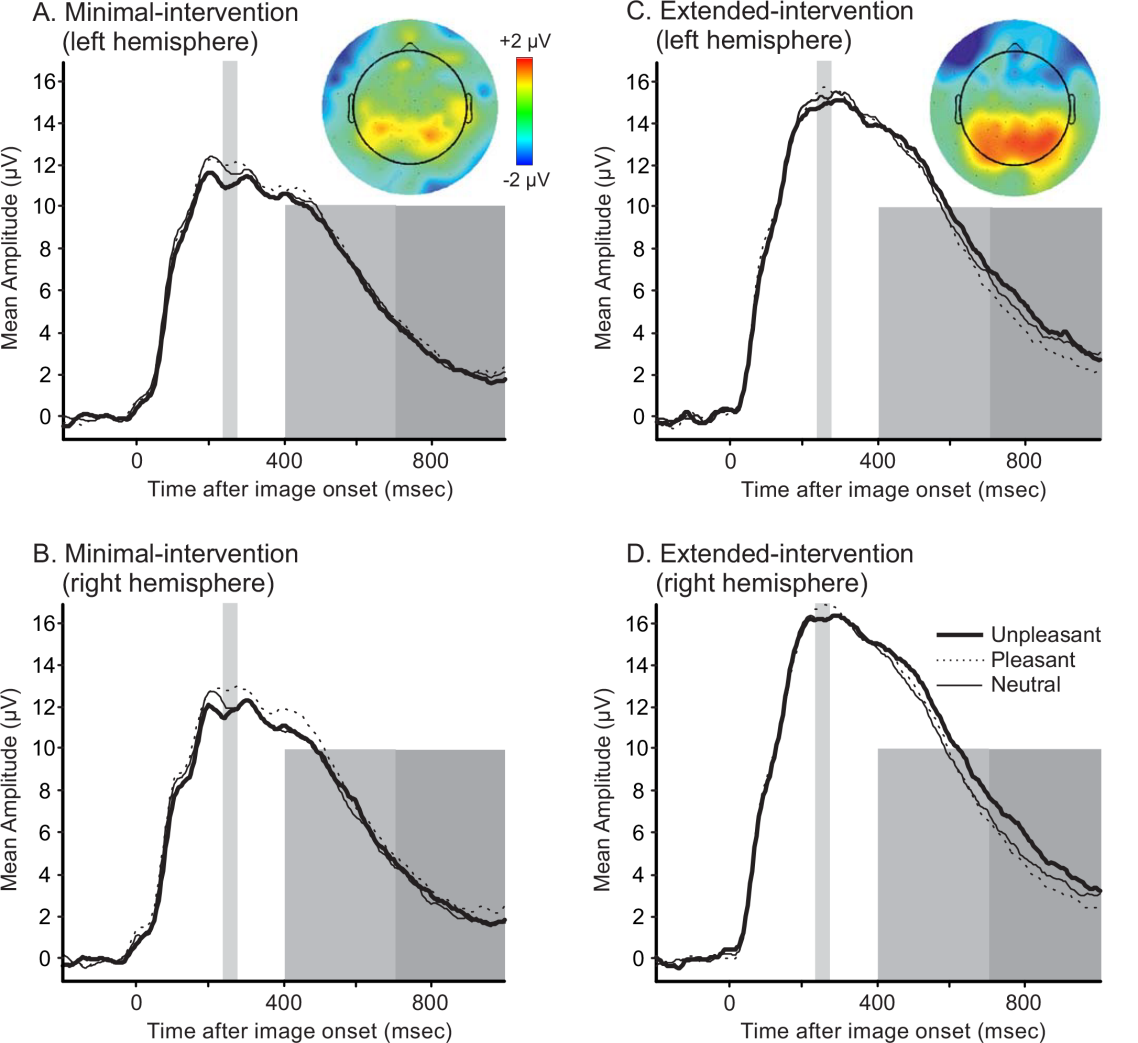
Fig 1A and 1C show ERPs for the left hemisphere (waveforms are from electrode O1 = electrode number 70). Fig 1B and 1D show ERPs for the right hemisphere (waveforms are from electrode O2 = electrode number 83). Waveforms are displayed from electrodes O1 and O2 because these electrodes are representative of both the EPN and the LPP components. The grey windows highlight the time windows used for analysis. For 1A–1D, the thin grey window represents the EPN. The thick light grey window represents the early LPP. The thick dark grey window represents the late LPP. Time windows used for each component are as follows: 240-280msec for the EPN; 700-1000msec for the early LPP; 700-1000msec for the late LPP. Difference topographies (activity related to unpleasant images minus other images) are shown for the window 700-1000msec after stimulus onset, as this is the window where minimal-intervention and extended-intervention groups differed. For both groups, the EPN was more negative following unpleasant images. The late LPP was modulated by valence for the extended-intervention group, but was not modulated by valence in the minimal-intervention group.

EPN amplitudes were more positive in the right than the left hemisphere (*F*(1,62) = 11.212, *p* = .001, *η*^*2*^ = .153). As shown in Fig 1, the EPN was influenced by valence (*F*(2,124) = 6.152, *p* = .003, *η*^*2*^ = .090). In line with previous findings, post-hoc contrasts confirmed that amplitudes were more negative for unpleasant images compared to neutral images (*p* = .002). Amplitudes did not differ between unpleasant and pleasant images (*p* = .338) nor between pleasant and neutral images (*p* = .153). No other main or interaction effects were statistically significant. The same results held if peak amplitudes were analysed (hemisphere main effect: *F*(1,62) = 8.334, *p* = .005, *η*^*2*^ = .118); valence main effect: (*F*(2,124) = 4.525, *p* = .013, *η*^*2*^ = .068).

### ERP Results: LPP

#### 400-700msec

Mean amplitudes in the period 400-700msec after stimulus onset differed according to image valence (*F*(2,124) = 4.422, *p* = .014, *η*^*2*^ = .067, see Fig 1). Post-hoc comparisons indicated that mean amplitudes were larger in response to unpleasant images versus neutral images (*p* = .006) but did not differ between unpleasant and pleasant images (*p* = .195) nor between pleasant and neutral images (*p* = .787). A main effect of hemisphere (*F*(1,62) = 6.079, *p* = .016, *η*^*2*^ = .089) and a hemisphere x group interaction effect (*F*(1,62) = 6.742, *p* = .012, *η*^*2*^ = .098), indicated that amplitudes did not differ between hemispheres for the minimal-intervention group (*p* = .927) but were larger in the right hemisphere for the extended-intervention group (*p* = .001). The main effect of group approached statistical significance (*F*(1,62) = 3.673, *p* = .060, *η*^*2*^ = .056). In other words, overall amplitudes appeared to be larger in the extended-intervention group than in the minimal-intervention group.

#### 700-1000msec

Mean amplitudes in the period 700-1000msec after stimulus onset differed according to image valence (*F*(2,124) = 9.869, *p* < .001, *η*^*2*^ = .137). This effect was qualified by an interaction between image valence and group (*F*(2,124) = 3.535, *p* = .032, *η*^*2*^ = .054). Specifically, the data indicated that the extended-intervention group was responsive to the emotional content of the images, but the minimal-intervention group was not. Post-hoc contrasts confirmed that amplitudes were larger in response to unpleasant images compared to neutral (*p* = .006) and pleasant (*p* < .001) images only for the extended-intervention group. Amplitudes did not differ according to image valence for the minimal-intervention group (unpleasant vs. neutral: *p* = .246; unpleasant vs. pleasant: *p* = .643). Late LPP amplitudes to pleasant and neutral images did not differ for either group (minimal-intervention: *p* = .973; extended-intervention: *p* = .144), presumably because highly arousing (erotic) images could not be included in the pleasant stimulus set due to the young age of the participants **[41]**. No other main or interaction effects were statistically robust.

### Relationships between ERP and Self-Report data

**Fig 2:**
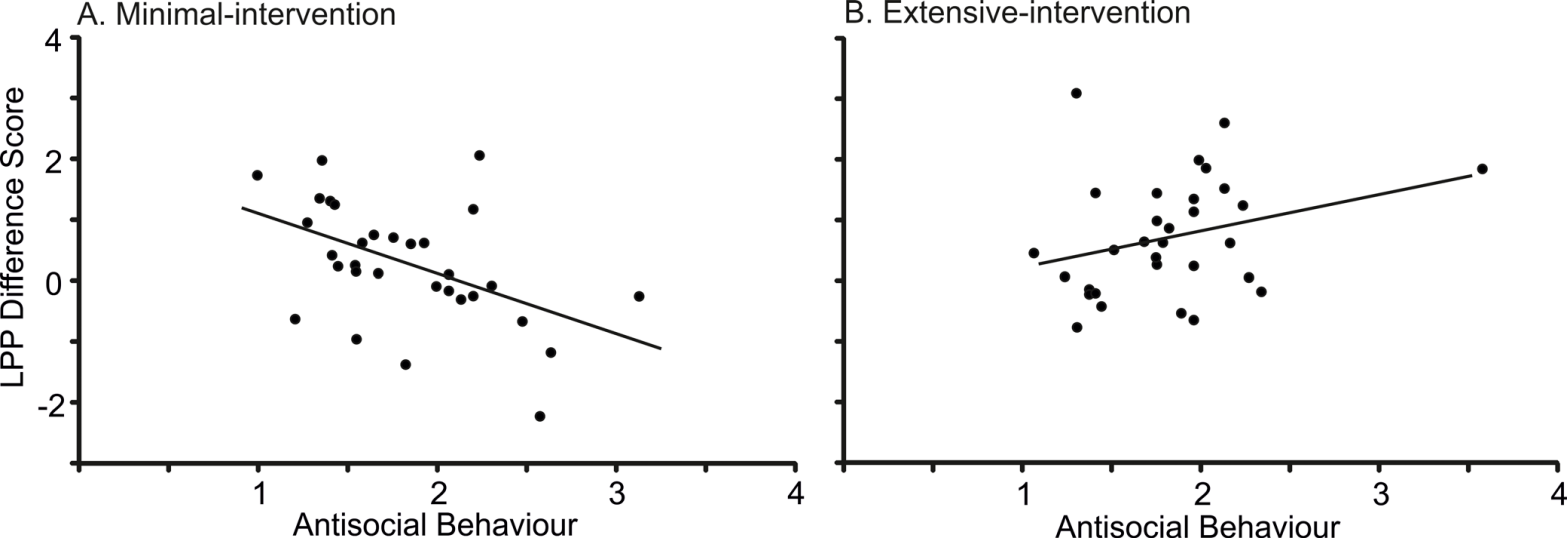
Correlations between antisocial behaviour scores (as measured by the Self Report of Youth Behaviour) and LPP difference scores (negative images minus other images, collapsed across the whole LPP window: 400-1000msec after stimulus onset). Note: Correlations are shown for the minimal-intervention group (A) and the extended-intervention group (B). Lower levels of antisocial behaviour were associated with better emotion processing in the minimal-intervention group only.

Given that emotional reactivity (as measured via the LPP) differed across groups, we investigated the relationships among LPP amplitude and participants’ self-report data. To this end, LPP difference scores were calculated for each participant (mean amplitudes for unpleasant images minus mean amplitudes averaged across neutral and pleasant images), computed for each LPP window (400-700msec and 700-1000msec), collapsed across hemispheres. For each group, the linear relationship between LPP difference scores and the self-report measures were assessed using Pearson’s correlations. As shown in Fig 2, strong negative correlations between antisocial behaviour (as indexed by the Self Report of Youth Behaviour) and LPP difference scores were evident for the minimal-intervention group. This relationship held across both time windows of the LPP: 400-700msec (*r* = -.484, *p* = .006) and 700-1000msec (*r* = -.414, *p* = .021). Removal of the outlier in the extended-intervention group did not alter the finding that the negative correlation between antisocial behaviour and emotion processing was restricted to the minimal-intervention group. In other words, lower levels of antisocial behaviour were associated with greater differentiation between unpleasant images and other images (larger LPP difference scores) for minimal-intervention participants. No other correlations were statistically significant.

Given that antisocial behaviour correlated with ERP difference amplitudes (at least for the minimal-intervention group), we tested whether differences in antisocial behaviour might account for the intervention effects reported above. Therefore, antisocial behaviour was entered as a covariate into the image valence x group ANOVA at 700-1000msec (the time window which revealed a significant image valence and group interaction). Even with antisocial behaviour as a covariate, this interaction was still significant (*F*(2,118) = 3.515, *p* = .033, *η*^*2*^ = .056), indicating that the obtained results cannot be accounted for by differences in antisocial behaviour.

## Discussion

The present study investigated whether electrophysiological differences exist between two groups of at-risk adolescent males: those who have received extensive psychosocial support and those who are new to treatment. The overarching aim was to provide preliminary evidence that electrophysiological markers of emotional responding are sensitive to therapeutic change in young people, and provide first evidence regarding the size of any such effects, in order to catalyse and inform further longitudinal investigations. Assuming that psychosocial intervention is associated with improved emotion processing, we hypothesised that emotional valence would impact the LPP in the extended-intervention group but not in the minimal-intervention group. Our major findings yielded evidence consistent with this hypothesis. The minimal-intervention participants showed attenuated neural responses to the emotional images, as the late LPP did not differentiate between image valances in that group. By contrast, the extended-intervention group showed LPP responses that were similar to those observed in studies of adults and children in low risk populations. These data extend work by Lewis et al. [42] and Woltering et al. [43], suggesting that ERPs may be useful as markers of therapeutic change and to delineate the neural mechanisms associated with such change. The findings must be considered preliminary because they are not based on random allocation, and hence we cannot rule out the possibility that undetected pre-existing differences between the groups were responsible for the effects we observed. Nevertheless, as a proof-of-principle, to be more formally tested in an RCT study, the findings are encouraging.

For both groups of participants, the EPN and the early LPP were modulated by valence in the direction typically observed in low-risk populations. Subjective valence ratings were also equivalent across the two groups, and paralleled adult normative ratings [32]. Therefore, despite the evidence suggesting emotional hypo-reactivity in the minimal-intervention group (via the late LPP), all participants appeared to have processed the emotional content of the images to some degree. Why might emotion processing be impaired for certain brain responses but not others? In order to answer this question, it is helpful to consider the functional significance of the different ERP components that were measured. As previously discussed, the EPN is one of the earliest components responsive to emotion, and reflects early visual perception. By contrast, the latency and topography of the early LPP window overlaps with the P3, and therefore likely indexes a later cognitive-affective process, such as increased transient attention towards emotional content or meaning (see arguments by [40]). The late LPP window likely represents higher order cognitive-affective processing, namely sustained attention towards emotional information [44–46]. It is therefore reasonable to argue that initial visual attention and perception were not abnormal in the minimal-intervention group. Instead, higher-order evaluative processing appeared to be impaired in at-risk adolescents who had received minimal psychosocial intervention, and might be understood as having normalised in those adolescents who had received intervention.

Although the EPN and the early LPP components were both sensitive to valence, these components differed in other respects. EPN amplitudes were more positive in the right hemisphere versus the left hemisphere. The observed hemispheric lateralisation may be explained by the ‘right hemisphere hypothesis’ of emotion processing. That hypothesis argues that–at least for facial stimuli–the right hemisphere is more strongly involved than the left hemisphere in processing emotional information [47–49]. Regarding the early LPP, a significant hemisphere x group interaction effect emerged. Specifically, amplitudes were more positive in the right hemisphere versus the left hemisphere for the extended-intervention group only. It could therefore be argued that participants in the extended-intervention group were able to exploit the specialisation of the right hemisphere to process emotional information. By contrast, this lateralisation was absent in young people who were new to the psycho-social intervention, whose difficulties may have impinged on their ability to process the emotional images.

Another interesting finding to emerge from the current study was the inverse relationship between emotional reactivity (as measured by LPP difference scores) and the severity of delinquent behaviour. This finding is in keeping with other studies showing that young people with externalizing problems show hypo-reactive neural and physiological responses to emotional stimuli [2, 11, 13, 43, 50]. The fact that the relationship between antisocial behaviour and neural activity was restricted to the minimal-intervention group is consistent with the idea that the emotional processing differed between the two participant groups.

In addition to reduced emotional reactivity, our data suggested a more generalised reduction in cortical activity for the minimal-intervention group (as evidenced by a trend towards smaller overall LPP amplitudes in the minimal intervention group–although this effect only approached significance). Reduced LPP magnitudes probably reflect the same cognitive mechanisms underlying reduced P3 amplitudes. Reduced P3 amplitudes have been reliably associated with various forms of psychopathology including disinhibitory disorders, alcoholism and dependency disorders, schizophrenia and depression [16, 51–54]. Importantly, increases in P3 amplitude (that is, normalisation of the P3 response) have followed successful therapeutic intervention–at least in adults suffering from depression [55]. The current data therefore extends the existing literature, and provides support for the notion that P3 amplitudes (and the cognitive skills that they reflect) are malleable and associated with therapeutic change.

The current study was able to address several critical alternative explanations of our findings. First, stimulus complexity was matched across image valence categories, thereby ruling out perceptual explanations of the observed results [19, 56]. Second, ERP differences between the minimal-intervention and extended-intervention groups were unlikely to have been driven by age or socio-economic status because these demographic features were matched across the groups. Third, the number of excluded trials was equivalent across groups and valence conditions indicating the results are unlikely to be due to differences in EEG signal noise.

Notwithstanding its benefits, this study has limitations that require acknowledgment. The study was designed as a ‘proof of principle’ investigation into the viability of the LPP as a marker of therapeutic change in at-risk adolescents. We therefore make no claims about causality. Causal conclusions would necessitate a randomised, prospective study design. Given the cross-sectional nature of the study, we were unable to reveal whether the young people’s symptoms and behaviour improved as a result of their participation in the psycho-social intervention. Further, we deliberately sampled a community based sample of high-risk adolescents rather than a discrete psychiatric population. This is because we were not interested in elucidating the specific mechanisms underlying abnormal brain processing in a discrete psychiatric condition. Rather, the current study was primarily concerned with establishing an evidence base for the use of the LPP as a biomarker in therapeutic research. In practice, adolescent intervention services typically accommodate clients with a diverse range of psychological needs (often involving social adversity), many of whom cannot be easily classified into DSM or ICD classifications of psychological illness. Indeed, this aspect of the current study could be considered a strength–the fact that we observed group differences in the LPP even with such a potentially heterogeneous group suggests that LPP effects should certainly be similar or even greater in studies with more homogeneous clinical groups. Finally, the exclusion of females is a limitation of our study. As a proof of principle study, the current investigation was restricted to male adolescents because at-risk male adolescents typically present differently to at-risk females. However, it would be very useful for future research to include both male and female participants.

To the best of our knowledge, the current study is the first to investigate the brain-basis of emotion processing in socially disadvantaged adolescents. It provides valuable insight into the potential utility of the LPP as a tool for assessing therapeutic change by revealing that emotional stimuli evoke distinct patterns of neural activation between adolescents who have received minimal versus extensive psychosocial intervention. These novel data therefore encourage the use of electrophysiology as a biomarker of therapeutic change longitudinal studies and formal RCTs, and it is anticipated that this work will lay the foundation stones for future work in this field.

## References

[1] Pincham HL, Bryce D, Pasco Fearon R. The neural correlates of emotion processing in juvenile offenders. Developmental Science. 2014 10.1111/desc.12262 25440113

[2] Sterzer P, Stadler C, Krebs A, Kleinschmidt A, Poustka F. Abnormal neural responses to emotional visual stimuli in adolescents with conduct disorder. Biological Psychiatry. 2005;57(1):7–15. 15607294 10.1016/j.biopsych.2004.10.008

[3] Gaffrey MS, Barch DM, Singer J, Shenoy R, Luby JL. Disrupted amygdala reactivity in depressed 4-to 6-year-old children. Journal of the American Academy of Child & Adolescent Psychiatry. 2013;52(7):737–46. 23800487 10.1016/j.jaac.2013.04.009 PMC3725819

[4] Gollan JK, Hoxha D, Chihade D, Pflieger ME, Rosebrock L, Cacioppo J. Frontal alpha EEG asymmetry before and after behavioral activation treatment for depression. Biological psychology. 2014;99:198–208. 10.1016/j.biopsycho.2014.03.003 24674708 PMC4609576

[5] Kaviani H, Gray J, Checkley S, Raven P, Wilson G, Kumari V. Affective modulation of the startle response in depression: influence of the severity of depression, anhedonia, and anxiety. Journal of affective disorders. 2004;83(1):21–31. 15546642 10.1016/j.jad.2004.04.007

[6] Fairchild G, Van Goozen SH, Stollery SJ, Goodyer IM. Fear conditioning and affective modulation of the startle reflex in male adolescents with early-onset or adolescence-onset conduct disorder and healthy control subjects. Biological Psychiatry. 2008;63(3):279–85. 17765205 10.1016/j.biopsych.2007.06.019

[7] Sebastian CL, McCrory EJ, Cecil CA, Lockwood PL, De Brito SA, Fontaine NM, et al Neural Responses to Affective and Cognitive Theory of Mind in Children With Conduct Problems and Varying Levels of Callous-Unemotional TraitsNeural Responses to Affective and Cognitive ToM. Archives of General Psychiatry. 2012;69(8):814–22. 10.1001/archgenpsychiatry.2011.2070 22868935

[8] Cecil CAM, Viding E, Barker ED, Guiney J, McCrory EJ. Double disadvantage: The influence of childhood maltreatment and community violence exposure on adolescent mental health. Journal of Child Psychology and Psychiatry. 2014. 10.1111/jcpp.12213 24611776

[9] Shaw DS, Shelleby EC. Early-Starting Conduct Problems: Intersection of Conduct Problems and Poverty. Annual Review of Clinical Psychology. 2014;10. 10.1146/annurev-clinpsy-032813-153650 PMC4194898 24471370

[10] Shonkoff JP, Garner AS. Committee on Psychosocial Aspects of Child and Family Health; Committee on Early Childhood, Adoption, and Dependent Care; Section on Developmental and Behavioral Pediatrics. The lifelong effects of early childhood adversity and toxic stress. Pediatrics. 2012;129(1):e232–e46. 10.1542/peds.2011-2663 22201156

[11] Fairchild G, Stobbe Y, van Goozen S, Calder AJ, Goodyer IM. Facial expression recognition, fear conditioning, and startle modulation in female subjects with conduct disorder. Biological Psychiatry. 2010;68(3):272–9. 10.1016/j.biopsych.2010.02.019 20447616 PMC2954286

[12] Passamonti L, Fairchild G, Goodyer IM, Hurford G, Hagan CC, Rowe JB, et al Neural abnormalities in early-onset and adolescence-onset conduct disorder. Archives of General Psychiatry. 2010;67(7):729–38. 10.1001/archgenpsychiatry.2010.75 20603454 PMC4471104

[13] van Goozen S, Snoek H, Matthys W, Rossum I, Engeland H. Evidence of fearlessness in behaviourally disordered children: a study on startle reflex modulation. Journal of Child Psychology and Psychiatry. 2004;45(4):884–92. 15056318 10.1111/j.1469-7610.2004.00280.x

[14] Raine A, Venables PH, Williams M. Relationships between central and autonomic measures of arousal at age 15 years and criminality at age 24 years. Archives of General Psychiatry. 1990;47(11):1003–7. 2241502 10.1001/archpsyc.1990.01810230019003

[15] Junghöfer M, Bradley MM, Elbert TR, Lang PJ. Fleeting images: a new look at early emotion discrimination. Psychophysiology. 2001;38(2):175–8. 11347862

[16] Schupp HT, Markus J, Weike AI, Hamm AO. Emotional facilitation of sensory processing in the visual cortex. Psychological science. 2003;14(1):7–13. 12564747 10.1111/1467-9280.01411

[17] Schupp HT, Öhman A, Junghöfer M, Weike AI, Stockburger J, Hamm AO. The facilitated processing of threatening faces: an ERP analysis. Emotion. 2004;4(2):189 15222855 10.1037/1528-3542.4.2.189

[18] Schupp HT, Flaisch T, Stockburger J, Junghöfer M. Emotion and attention: event-related brain potential studies. Progress in brain research. 2006;156:31–51. 17015073 10.1016/S0079-6123(06)56002-9

[19] Bradley MM, Hamby S, Löw A, Lang PJ. Brain potentials in perception: picture complexity and emotional arousal. Psychophysiology. 2007;44(3):364–73. 17433095 10.1111/j.1469-8986.2007.00520.x

[20] Cuthbert BN, Schupp HT, Bradley MM, Birbaumer N, Lang PJ. Brain potentials in affective picture processing: covariation with autonomic arousal and affective report. Biological psychology. 2000;52(2):95–111. 10699350 10.1016/s0301-0511(99)00044-7

[21] Naumann E, Bartussek D., Diedrich O., Laufer M.E. Assessing cognitive and affective information processing functions of the brain by means of the late positive complex of the event-related potential. Journal of Psychophysiology. 1992;6(4):285–98.

[22] Lang PJ, Bradley MM, Cuthbert BN. International affective picture system (IAPS): Technical manual and affective ratings Gainesville, FL: NIMH Center for the Study of Emotion and Attention, University of Florida; 1997.

[23] Lang PJ, Bradley MM, Fitzsimmons JR, Cuthbert BN, Scott JD, Moulder B, et al Emotional arousal and activation of the visual cortex: an fMRI analysis. Psychophysiology. 1998;35(2):199–210. 9529946

[24] Schupp HT, Junghöfer M, Weike AI, Hamm AO. The selective processing of briefly presented affective pictures: An ERP analysis. Psychophysiology. 2004;41(3):441–9. 15102130 10.1111/j.1469-8986.2004.00174.x

[25] Liu Y, Huang H, McGinnis-Deweese M, Keil A, Ding M. Neural substrate of the late positive potential in emotional processing. The Journal of Neuroscience. 2012;32(42):14563–72. 10.1523/JNEUROSCI.3109-12.2012 23077042 PMC3516184

[26] Gaskell C. Kids Company helps with the whole problem London: Kids Company; 2008.

[27] Jovchelovitch S, Concha N. Kids Company: a diagnosis of the organisation and its interventions. 2013.

[28] Cho H, Hallfors DD, Sánchez V. Evaluation of a high school peer group intervention for at-risk youth. Journal of abnormal child psychology. 2005;33(3):363–74. 15957563 10.1007/s10802-005-3574-4

[29] Mills RC, Dunham RG, Alpert GP. Working with high-risk youth in prevention and early intervention programs: toward a comprehensive wellness model. Adolescence. 1988. 3057818

[30] Podorefsky DL, McDonald-Dowdell M, Beardslee WR. Adaptation of preventive interventions for a low-income, culturally diverse community. Journal of the American Academy of Child & Adolescent Psychiatry. 2001;40(8):879–86. 11501686 10.1097/00004583-200108000-00008

[31] KidsCompany. Key Worker Tool Kit. London: Kids Company; 2010.

[32] Lang PJ, Bradley MM, Cuthbert BN. International affective picture system (IAPS): Affective ratings of pictures and instruction manual: University of Florida, Gainesville, FL; 2008.

[33] McManis MH, Bradley MM, Berg WK, Cuthbert BN, Lang PJ. Emotional reactions in children: Verbal, physiological, and behavioral responses to affective pictures. Psychophysiology. 2001;38(2):222–31. 11347868

[34] Schupp HT, Cuthbert BN, Bradley MM, Cacioppo JT, Ito T, Lang PJ. Affective picture processing: the late positive potential is modulated by motivational relevance. Psychophysiology. 2000;37(2):257–61. 10731776

[35] Weinberg A, Hajcak G. Beyond good and evil: the time-course of neural activity elicited by specific picture content. Emotion. 2010;10(6):767 10.1037/a0020242 21058848

[36] Goodman R. The Strengths and Difficulties Questionnaire: a research note. Journal of Child Psychology and Psychiatry. 1997;38(5):581–6. 9255702 10.1111/j.1469-7610.1997.tb01545.x

[37] Olweus D. Prevalence and incidence in the study of antisocial behavior: definitions and measurements In: Klein MW, editor. Cross-national research in self-reported crime and delinquency. Dordrecht, Netherlands: Kluwer-Nijhoff; 1989 p. 187–201.

[38] Jessor R, Jessor SL. Problem behavior and psychosocial development: A longitudinal study of youth New York: Academic Press; 1977.

[39] Nolan H, Whelan R, Reilly R. FASTER: Fully Automated Statistical Thresholding for EEG artifact Rejection. Journal of Neuroscience Methods. 2010;192(1):152–62. 10.1016/j.jneumeth.2010.07.015 20654646

[40] Hajcak G, Dennis TA. Brain potentials during affective picture processing in children. Biological psychology. 2009;80(3):333–8. 10.1016/j.biopsycho.2008.11.006 19103249 PMC2656402

[41] De Cesarei A, Codispoti M. When does size not matter? Effects of stimulus size on affective modulation. Psychophysiology. 2006;43(2):207–15. 16712591 10.1111/j.1469-8986.2006.00392.x

[42] Lewis MD, Granic I, Lamm C, Zelazo PD, Stieben J, Todd RM, et al Changes in the neural bases of emotion regulation associated with clinical improvement in children with behavior problems. Development and psychopathology. 2008;20(03):913–39. 18606038 10.1017/S0954579408000448

[43] Syngelaki EM, Fairchild G, Moore SC, Savage JC, van Goozen SHM. Affective startle potentiation in juvenile offenders: The role of conduct problems and psychopathic traits. Social Neuroscience. 2013;8(2):112–21. 10.1080/17470919.2012.712549 22856454

[44] Pastor MC, Bradley MM, Löw A, Versace F, Moltó J, Lang PJ. Affective picture perception: Emotion, context, and the late positive potential. Brain research. 2008;1189:145–51. 18068150 10.1016/j.brainres.2007.10.072 PMC2993239

[45] Codispoti M, Ferrari V, Bradley MM. Repetitive picture processing: Autonomic and cortical correlates. Brain research. 2006;1068(1):213–20. 16403475 10.1016/j.brainres.2005.11.009

[46] Codispoti M, Ferrari V, Bradley MM. Repetition and event-related potentials: Distinguishing early and late processes in affective picture perception. Journal of Cognitive Neuroscience. 2007;19(4):577–86. 17381249 10.1162/jocn.2007.19.4.577

[47] Gainotti G. Emotional behavior and hemispheric side of the lesion. Cortex. 1972;8(1):41–55. 5031258 10.1016/s0010-9452(72)80026-1

[48] Levy J, Heller W, Banich MT, Burton LA. Asymmetry of perception in free viewing of chimeric faces. Brain and cognition. 1983;2(4):404–19. 6546034 10.1016/0278-2626(83)90021-0

[49] Prete G, Laeng B, Fabri M, Foschi N, Tommasi L. Right hemisphere or valence hypothesis, or both? The processing of hybrid faces in the intact and callosotomized brain. Neuropsychologia. 2015;68:94–106. 10.1016/j.neuropsychologia.2015.01.002 25575451

[50] Syngelaki EM, Fairchild G, Moore SC, Savage JC, van Goozen SHM. Fearlessness in juvenile offenders is associated with offending rate. Developmental Science. 2013;16(1):84–90. 10.1111/j.1467-7687.2012.01191.x 23278929

[51] Bauer LO, Hesselbrock VM. P300 decrements in teenagers with conduct problems: implications for substance abuse risk and brain development. Biological Psychiatry. 1999;46(2):263–72. 10418702 10.1016/s0006-3223(98)00335-7

[52] Gao Y, Raine A, Venables PH, Mednick SA. The Association Between P3 Amplitude at Age 11 and Criminal Offending at Age 23. Journal of Clinical Child & Adolescent Psychology. 2013;42(1):120–30. 22963083 10.1080/15374416.2012.719458 PMC4166541

[53] Hicks BM, Bernat E, Malone SM, Iacono WG, Patrick CJ, Krueger RF, et al Genes mediate the association between P3 amplitude and externalizing disorders. Psychophysiology. 2007;44(1):98–105. 17241145 10.1111/j.1469-8986.2006.00471.x PMC2365473

[54] Iacono WG, Carlson SR, Malone SM, McGue M. P3 event-related potential amplitude and the risk for disinhibitory disorders in adolescent boys. Archives of General Psychiatry. 2002;59(8):750 12150652 10.1001/archpsyc.59.8.750

[55] Naga Venkatesha Murthy P, Gangadhar B, Janakiramaiah N, Subbakrishna D. Normalization of P300 amplitude following treatment in dysthymia. Biological Psychiatry. 1997;42(8):740–3. 9325569 10.1016/s0006-3223(97)00296-5

[56] Ito TA, Cacioppo JT. Electrophysiological evidence of implicit and explicit categorization processes. Journal of Experimental Social Psychology. 2000;36(6):660–76.

